# The rs10781468 Genetic Polymorphism of the Gαq (GNAQ) Gene Is Associated With Responsiveness to Opioid Analgesics in Patients With Postoperative and Cancer Pain: An Exploratory Study

**DOI:** 10.1002/npr2.70148

**Published:** 2026-06-30

**Authors:** Hiroaki Owada, Daisuke Nishizawa, Tsutomu Mieda, Miki Tsujita, Hideyuki Nakagawa, Shigeki Yamaguchi, Rikuhei Tsuchida, Yaeko Yokoshima, Akira Kitamura, Masakazu Hayashida, Ken‐ichi Fukuda, Tatsuya Ichinohe, Kazutaka Ikeda, Makoto Kurano, Masahiko Sumitani, Kanji Uchida

**Affiliations:** ^1^ Department of Anesthesiology and Pain Relief Center The University of Tokyo Hospital Bunkyo‐ku Tokyo Japan; ^2^ Addictive Substance Project Tokyo Metropolitan Institute of Medical Science Setagaya‐ku Tokyo Japan; ^3^ Department of Neuropsychopharmacology National Institute of Mental Health, National Center of Neurology and Psychiatry Kodaira‐shi Tokyo Japan; ^4^ Department of Anesthesiology Saitama Medical University Hospital Saitama‐shi Saitama Japan; ^5^ Department of Anesthesiology Saitama Medical University International Medical Center Hidaka‐shi Saitama Japan; ^6^ Division of Colorectal Surgery, Department of Surgery Tokyo Women's Medical University Shinjuku‐ku Tokyo Japan; ^7^ Department of Anesthesiology and Pain Medicine Juntendo University Hospital Bunkyo‐ku Tokyo Japan; ^8^ Department of Oral Health and Clinical Science Tokyo Dental College Chiyoda‐ku Tokyo Japan; ^9^ Department of Dental Anesthesiology Tokyo Dental College Chiyoda‐ku Tokyo Japan; ^10^ Department of Clinical Laboratory Medicine The University of Tokyo Hospital Bunkyo‐ku Tokyo Japan; ^11^ Department of Pain and Palliative Medicine The University of Tokyo Hospital Bunkyo‐ku Tokyo Japan

**Keywords:** cancer pain, genetic polymorphism, GNAQ, opioid responsiveness, pain management, postoperative pain

## Abstract

**Background:**

G protein–coupled receptors, the largest class of cell surface receptors, are ubiquitously expressed throughout the body. Different subtypes of G protein α subunits activate distinct intracellular signaling pathways and mediate opioid analgesic effects. However, the contributions of these Gα subunits remain unclear. We conducted a hypothesis‐generating, two‐stage candidate gene association study to identify potential genetic loci related to pain processing and opioid analgesia, focusing on genes encoding G protein α subunits.

**Methods:**

To identify candidate single‐nucleotide polymorphisms (SNPs) in seven genes encoding G protein α subunits, we analyzed postoperative opioid consumption, pain intensity, and opioid responsiveness in two exploratory cohorts [laparoscopy‐assisted colectomy (*n* = 350) and mandibular plastic surgery (*n* = 354)]. For identified candidate SNPs, we further evaluated opioid responsiveness in a confirmatory cohort of 89 patients with cancer pain. We also conducted a preliminary sensitivity analysis adjusting for relevant covariates.

**Results:**

Two SNPs (rs10781468 in the *GNAQ* gene and rs308049 in the *GNA11* gene) showed significant associations in both exploratory cohorts. Patients with postoperative pain who were homozygous for the minor allele of rs10781468 exhibited higher opioid sensitivity or lower pain severity. In the confirmatory cohort, homozygosity for the major allele of rs10781468 was significantly associated with increased opioid sensitivity for cancer pain management under the dominant model (*p* = 0.025) and the genotypic model (*p* = 0.020), whereas rs308049 was not. The observed phenotypes and associated genetic models differed across cohorts, and sensitivity analyses continued to demonstrate some significant associations.

**Conclusions:**

As a hypothesis‐generating investigation, we suggest that rs10781468 may serve as a potential candidate marker of responsiveness to opioid analgesics. Our findings should be validated in a large‐scale study with a homogeneous pain type in which the opioid administration protocol is controlled to further examine the association observed in this exploratory study.

## Abstract 2

In our exploratory hypothesis‐generating study using patients with postoperative pain and cancer pain, for rs10781468 in the *GNAQ* gene, the minor allele was associated with reduced opioid consumption and an increased pain threshold.

## Graphical abstract

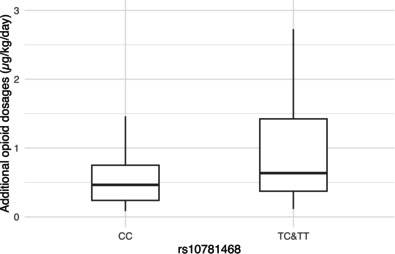

## 1 Introduction

Pain is classified into three types: nociceptive, neuropathic, and nociplastic pain. These categories differ in their underlying pathophysiological mechanisms and, therefore, in their responses to opioid analgesics. Among multiple biological factors, G protein–coupled receptors (GPCRs) are widely recognized to influence both opioid sensitivity and pain severity. A representative example is that opioid receptors themselves are GPCRs.

GPCRs represent the largest class of cell‐surface receptors in humans and play a key role in drug development as major therapeutic targets [1]. Upon ligand binding, GPCRs activate heterotrimeric G proteins, which consist of an α subunit and a βγ dimer, thereby initiating downstream intracellular signaling. The α subunit largely determines the signaling pathway engaged and is classified into four main subtypes: Gα_q/11_, Gα_12/13_, Gα_i/o_, and Gα_s_. Each subtype triggers distinct intracellular signaling pathways, resulting in specific physiological effects.

To explain individual differences in responses to GPCR agonists or antagonists, previous genetic studies have generally focused on receptor‐level variations, such as genetic polymorphisms in opioid receptors [2]. To our knowledge, only one study has examined individual differences in opioid signaling at the level of G protein subunits and reported that a genetic polymorphism in the β subunit gene influenced the intensity of withdrawal in opioid dependence [3]. Relatively little attention has been given to genetic variation in G protein α subunits, despite their central role in initiating intracellular signaling from numerous GPCRs. Several genetic polymorphisms in G protein α subunit genes (*GNAQ* and *GNA11*) have been reported to be associated with clinical phenotypes such as cardiovascular morbidity, calcium abnormalities, and insulin resistance [4, 5]. Clarifying the contribution of G protein α subunit genetic polymorphisms to opioid sensitivity and pain perception may provide novel insights into the intracellular mechanisms underlying diverse pain phenotypes.

To our knowledge, no study has examined the relationship between G protein α subunit genetic polymorphisms and opioid sensitivity and pain intensity. To explore this hypothesized relationship, we investigated the seven main G protein α subunit subtypes without focusing on specific GPCRs in order to examine their genetic associations with pain intensity and opioid sensitivity. As a hypothesis‐generating investigation, we conducted a two‐stage study: first, candidate single‐nucleotide polymorphisms (SNPs) were identified in a cohort with postoperative nociceptive pain; second, these SNPs were evaluated in a cancer pain cohort, which typically involves more complex pain phenotypes, to assess their associations with opioid sensitivity and pain intensity.

## 2 Methods

### 2.1 Ethics Statement

The institutional review board at each participating hospital approved the study protocol (G2804), and all participants provided written informed consent. This study was registered in the University Medical Information Network (UMIN; trial ID: UMIN000008595).

### 2.2 Experiment 1

We analyzed the relationships between nociceptive pain intensity and genetic polymorphisms in *GNAQ*, *GNA11*, *GNA12*, *GNA13*, *GNAI1*, *GNAO1*, and *GNAS*, which encode Gα_q/11_, Gα_12/13_, Gα_i/o_, and Gα_s_, respectively. We investigated these associations using data from two cohorts derived from our previous genetic polymorphism studies [6, 7]: laparoscopy‐assisted colorectal and mandibular plastic surgery.

#### 2.2.1 Laparoscopy‐Assisted Colorectal Surgery

In the first exploratory cohort [6], we enrolled 351 adult patients without severe coexisting systemic disease (American Society of Anesthesiologists Physical Status [ASA‐PS] I or II) who underwent scheduled laparoscopy‐assisted colectomy (LAC) for colon or rectal cancer at Saitama Medical University International Medical Center. Patients with severe coexisting disease (ASA‐PS III or higher), those receiving analgesics for chronic pain, and those who were unlikely to be able to use a patient‐controlled analgesia pump were excluded. Intravenous patient‐controlled analgesia (IV‐PCA) was used for postoperative pain management. The IV‐PCA was set to deliver a bolus dose of fentanyl 20 μg with a lockout interval of 5 min, without continuous infusion. Postoperative fentanyl consumption was recorded during the 24‐h postoperative period (postoperative opioid consumption). If IV‐PCA alone failed to provide adequate analgesia, flurbiprofen and pentazocine were administered as rescue analgesics. Postoperative pain intensity was assessed using an 11‐point numerical rating scale (NRS) (0 = no pain; 10 = worst pain imaginable) 24 h after surgery.

#### 2.2.2 Mandibular Plastic Surgery

For the second exploratory cohort [7], we enrolled 355 healthy patients (ASA‐PS I) who underwent scheduled mandibular sagittal split ramus osteotomy (SSRO) for mandibular prognathism at Tokyo Dental College Suidoubashi Hospital. Patients with chronic pain, those receiving analgesics, and those with a history of Raynaud's phenomenon were excluded. The cold pressor–induced pain test was performed immediately before the induction of general anesthesia. In this test, withdrawal latency was recorded as the time to the first perception of pain after the participant's hand was immersed in a bucket of ice water. Pain perception latency (PPL) was measured twice: before and 3 min after an intravenous bolus administration of fentanyl (2.5 μg/kg) (pre‐PPL and post‐PPL). The difference in PPL (ΔPPL = post‐PPL−pre‐PPL) was used as an index of opioid sensitivity. After the cold pressor–induced test, general anesthesia was induced, IV‐PCA was initiated for postoperative pain management, and total fentanyl consumption during the 24‐h postoperative period was recorded. Postoperative pain intensity was assessed using a 100‐mm visual analog scale 24 h postoperatively. Fentanyl consumption in both cohorts was normalized to body weight and analyzed in relation to genetic polymorphisms.

#### 2.2.3 Genotyping Method

Due to incomplete data and failure to meet quality control criteria, a total of 350 and 354 DNA samples from patients who underwent LAC and SSRO, respectively, were used for genotyping. Genomic DNA was extracted from whole‐blood samples using standard procedures. The extracted DNA was dissolved in TE buffer (10 mM Tris–HCl and 1 mM ethylenediaminetetraacetic acid, pH 8.0). DNA concentration was adjusted to 100 ng/μL for whole‐genome genotyping using a NanoDrop ND‐1000 spectrophotometer (NanoDrop Technologies, Wilmington, DE, USA). Briefly, whole‐genome genotyping was performed using the Infinium assay II with an iScan system (Illumina, San Diego, CA, USA) according to the manufacturer's instructions. Genotyping was conducted using Illumina BeadChips—two types in the LAC cohort and five types in the SSRO cohort (LAC cohort: HumanOmniExpressExome‐8 v1.0 [256 samples, 951 117 markers] and v1.1 [95 samples, 958 178 markers]; SSRO cohort: HumanHap300 [40 samples, 317 503 markers], HumanHap300‐Duo [67 samples, 318 237 markers], Human610‐Quad v1 [6 samples, 620 901 markers], Human1M v1.0 [119 samples, 1 072 820 markers], and Human1M‐Duo v3 [123 samples, 1 199 187 markers]). The quality control of the genotyped data was conducted as described in previous reports [6, 7]. Briefly, whole‐genome genotyping data were evaluated using GenomeStudio with the Genotyping Module v3.3.7 (Illumina). Genotype data obtained from multiple BeadChips were merged to enable simultaneous analysis of all samples within each cohort. During data cleaning in both cohorts, samples with a genotype call rate of less than 0.95 were excluded from further analyses. As a result, one sample was excluded from the SSRO cohort. Markers with a genotype call frequency of less than 0.95 or a “cluster separation” value (i.e., an index of genotype cluster separation) of less than 0.1 were excluded from subsequent association analyses. Markers were not excluded on the basis of minor allele frequency values or Hardy–Weinberg equilibrium test results at the initial stage; however, Hardy–Weinberg equilibrium tests were conducted for the two most promising candidate SNPs (rs10781468 in the *GNAQ* gene and rs308049 in the *GNA11* gene) to confirm the absence of significant deviation from theoretical Hardy–Weinberg equilibrium.

Whole‐genome genotyping data were then used to analyze 211 SNPs in the LAC cohort and 77 SNPs in the SSRO cohort within the target gene regions (LAC cohort: *GNAQ*, 42; *GNA11*, 11; *GNA12*, 33; *GNA13*, 5; *GNAI1*, 27; *GNAO1*, 42; *GNAS*, 51 SNPs; SSRO cohort: *GNAQ*, 17; *GNA11*, 7; *GNA12*, 14; *GNA13*, 3; *GNAI1*, 9; *GNAO1*, 16; *GNAS*, 11 SNPs).

### 2.3 Experiment 2

#### 2.3.1 Participants

As a confirmatory cohort, we used data from our previous genetic polymorphism study [8]. Briefly, we enrolled 90 adult patients who were diagnosed with cancer pain, had a mean NRS score greater than 3 during the past week, and had a pain duration longer than 1 week at cancer pain‐research consortium hospitals [8] which are represented by The University of Tokyo Hospital. Patients with mild or more severe cognitive dysfunction, those with brain metastases, and those suspected of having pain originating from causes other than cancer itself were excluded. Daily opioid doses and pain intensity were recorded using an 11‐point NRS (pre‐NRS). Subsequently, 72 patients received additional opioid doses required for pain relief, and cancer pain intensity (post‐NRS) was reassessed. Opioid doses were converted to intravenous fentanyl equivalents and normalized to body weight. Total opioid doses (baseline and additional opioids) were recorded in these 72 patients. The percentage improvement in NRS (NRS improvement [%]) was calculated using the following formula: NRS improvement (%) = (pre‐NRS−post‐NRS) × 100/pre‐NRS.

#### 2.3.2 Genotyping Method

Due to incomplete data, a total of 89 samples were used for genotyping. Genomic DNA was extracted from whole‐blood samples using standard procedures.

Genomic DNA was whole‐genome amplified, fragmented, denatured, and hybridized in the same manner as in Experiment 1, and all samples were genotyped using the Omni1‐Quad BeadChip (Illumina; total markers: 1140419). Normalized bead intensity data for each sample were loaded into GenomeStudio software (Genotyping Module v1.8.4; Illumina), which converted fluorescence intensities into SNP genotypes. SNPs with a call frequency of less than 95%, deviation from Hardy–Weinberg equilibrium at a type I error level of less than 10^−3^, or a minor allele frequency of less than 10^−3^ were excluded. Whole‐genome genotyping data were then used to analyze the candidate SNPs identified in the first‐stage analysis.

### 2.4 Statistical Analysis

A two‐stage association analysis was conducted using the two postoperative pain cohorts for the first‐stage exploratory study and the cancer pain cohort for the second‐stage confirmatory study. In the exploratory stage, we analyzed the associations between SNPs in the *GNAQ*, *GNA11*, *GNA12*, *GNA13*, *GNAI1*, *GNAO1*, and *GNAS* genes and various opioid‐related outcomes. Specifically, we examined postoperative opioid consumption in both the LAC and SSRO cohorts and evaluated the correlation between pain intensity and opioid sensitivity (ΔPPL) in the SSRO cohort. To identify SNPs potentially associated with opioid sensitivity, nonparametric rank‐based analyses were performed, in which the phenotypic and genotypic data for each SNP were incorporated as dependent and independent variables, respectively. Because limited evidence links these genetic factors to opioid sensitivity, additive, dominant, and recessive genetic models were applied. Owing to the preliminary exploratory nature of this study, multiplicity was not considered in the analyses of the exploratory cohorts. SNPs with *p*‐values < 0.05 in both exploratory cohorts under the same genetic model were selected as candidate SNPs for the subsequent confirmatory study. As part of the sensitivity analysis, a multivariable linear regression analysis was conducted adjusting for covariates such as age and sex. For the candidate SNPs, we examined whether they were associated with opioid requirements and NRS scores for cancer pain using nonparametric statistical tests. The Jonckheere–Terpstra test was applied under an additive model (monotonic increase or decrease according to the number of minor allele copies). The Mann–Whitney *U* test was used for the dominant (one or two vs. zero copies of the minor allele) and recessive (two vs. zero or one copy of the minor allele) models, whereas the Kruskal–Wallis test followed by a post hoc Bonferroni correction was used for the genotypic model (zero vs. one vs. two copies of the minor allele). Because the present study was preliminary and hypothesis‐generating, multiplicity was not adjusted for in the respective analyses; statistical significance was defined as *p* < 0.05 for all tests. For SNPs that reached significance in the confirmatory cohort, the false discovery rate (FDR) was additionally calculated using the Benjamini–Hochberg step‐up procedure to account for multiple testing [9]. A threshold of 20% FDR was used to indicate robustness of associations [10]. Additionally, as part of the sensitivity analysis, a multivariable linear regression analysis was performed adjusting for age, sex, and baseline opioid doses. These analyses were evaluated according to genotype order (major homozygotes, heterozygotes, and minor homozygotes). All statistical analyses were performed using R version 4.3.1 (R Foundation for Statistical Computing, Vienna, Austria) and the Statistical Package for the Social Sciences (SPSS) version 22.0 (IBM Inc., Chicago, IL, USA).

## 3 Result

### 3.1 Experiment 1

In the LAC cohort, 22 of 211 SNPs in the *GNAQ*, *GNA11*, *GNA12*, *GNA13*, *GNAI1*, *GNAO1*, and *GNAS* genes were significantly associated with postoperative opioid consumption. In the SSRO cohort, 22 of 77 SNPs showed significant associations with ΔPPL, and 13 of 77 SNPs were significantly associated with postoperative opioid consumption. Among these significant SNPs, only two (rs10781468 in the *GNAQ* gene and rs308049 in the *GNA11* gene) demonstrated significant associations with the evaluated outcomes under the same genetic model in both postoperative pain cohorts. For rs10781468, postoperative opioid consumption was significantly decreased under the additive and recessive models in the LAC cohort (Table 1). Postoperative pain intensity was also significantly decreased under the recessive model (Table 1). In the SSRO cohort, ΔPPL was significantly increased under the additive, dominant, and recessive models (Table 2). For rs308049, postoperative opioid consumption was significantly decreased in both cohorts: under the additive model in the LAC cohort and under the additive and recessive models in the SSRO cohort (Tables 1 and 2). These two SNPs were subsequently advanced to the confirmatory stage.

**TABLE 1:** Associations of rs10781468 in the *GNAQ* gene and rs308049 in the *GNA11* gene polymorphisms with postoperative opioid consumption and pain intensity in patients receiving laparoscopic‐assisted colectomy (Experiment 1). Pain intensity was measured using the numerical rating scale (NRS) at postoperative 24 h. Jonckheere–Terpstra test was used for the additive model, and the Mann–Whitney *U* test was used for the recessive and dominant model.

Gene	SNPs	Genotype	Laparoscopy‐assisted colectomy (LAC) cohort						
			*n* (female)	Postoperative opioid consumption (μg/kg)			Postoperative pain intensity (NRS)		
				(Median (IQR))	Model	*p*	(Median (IQR))	Model	*p*
*GNAQ*	rs10781468 C>T	CC	118 (47)	10.5 (6.14–13.2)	Additive	0.049	2 (1–3)	Additive	0.103
		TC	180 (67)	9.17 (5.33–14.0)	Recessive	0.040	2 (1–3)	Recessive	0.006
		TT	52 (19)	7.87 (4.29–12.3)	Dominant	0.382	1 (0–2.38)	Dominant	0.643
*GNA11*	rs308049 C>T	CC	249 (89)	9.11 (5.25–13.3)	Additive	0.042	2 (1–3)	Additive	0.482
		TC	90 (39)	10.3 (5.51–15.3)	Recessive	0.051	2 (1–3)	Recessive	0.442
		TT	10 (4)	12.9 (9.91–16.0)	Dominant	0.161	2.5 (1–4.25)	Dominant	0.527

**TABLE 2:** Associations of rs10781468 in the *GNAQ* gene and rs308049 in the *GNA11* gene polymorphisms with prolongation of pain latencies immediately after opioid administration (PPLpost‐pre), postoperative opioid consumption, and pain intensity in patients receiving mandibular sagittal split ramus osteotomy (Experiment 1). Pain intensity was measured using visual analog scale (VAS) at postoperative 24 h. Jonckheere–Terpstra test was used for the additive model, and Mann–Whitney *U* test was used for the recessive and dominant models.

Gene	SNP	Genotype	Mandibular sagittal split ramus osteotomy (SSRO) cohort									
			*n* (female)	Prolongation of pain latencies immediately after opioid administration			Postoperative opioid consumption (μg/kg)			Postoperative pain intensity (VAS)		
				(Median (IQR))	Model	*p*	(Median (IQR))	Model	*p*	(Median (IQR))	Model	*p*
*GNAQ*	rs10781468 C>T	CC	117 (78)	10.0 (3.00–33.0)	Additive	0.008	2.18 (1.15–3.93)	Additive	0.088	25.0 (9.00–45.0)	Additive	0.454
		TC	173 (109)	11.0 (5.00–33.0)	Recessive	0.024	2.31 (1.00–4.08)	Recessive	0.074	24.0 (10.0–40.0)	Recessive	0.138
		TT	64 (43)	18.0 (6.75–40.0)	Dominant	0.042	2.49 (1.12–5.26)	Dominant	0.400	33.0 (11.8–42.0)	Dominant	0.970
*GNA11*	rs308049 C>T	CC	237 (151)	11.0 (4.00–33.0)	Additive	0.589	2.27 (1.08–4.05)	Additive	0.041	25.0 (10.0–40.0)	Additive	0.860
		TC	107 (73)	13.0 (4.50–39.5)	Recessive	0.551	2.17 (0.990–4.28)	Recessive	0.037	25.0 (10.0–42.8)	Recessive	0.477
		TT	10 (6)	10.5 (4.50–14.0)	Dominant	0.759	4.81 (2.45–6.40)	Dominant	0.760	30.5 (14.3–49.5)	Dominant	0.927

Additionally, for the two SNPs (rs10781468 and rs308049) that were significant in Experiment 1, sensitivity analyses adjusting for age and sex yielded the following results: in the LAC cohort, most associations were no longer significant, although some new associations reached significance (Table S1). In the SSRO cohort, most associations remained significant (Table S1).

### 3.2 Experiment 2

**FIGURE 1:**
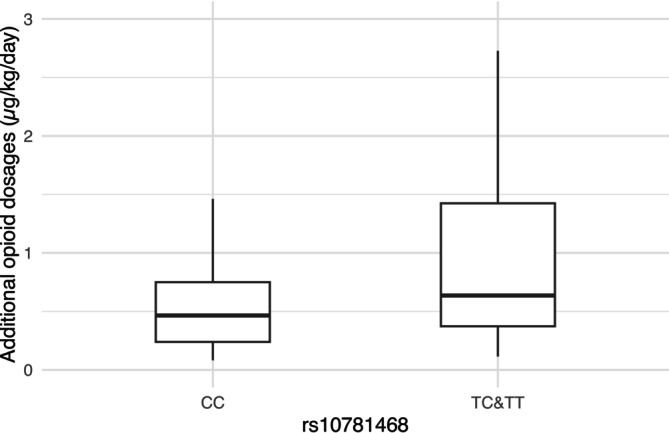
Box plot comparing additional opioid doses among rs10781468 genotypes. Patients homozygous for the major allele (CC) required significantly lower additional opioid doses than heterozygotes (TC) and minor allele homozygotes (TT), as indicated by the Mann–Whitney *U* test (*p* < 0.05). In the box plot, boxes represent the interquartile range (IQR), and the horizontal line indicates the median. Whiskers extend to the minimum and maximum values within 1.5 × IQR.

Of the two candidate SNPs (rs10781468 and rs308049), rs10781468 showed no significant associations with any outcomes before therapeutic intervention or with post‐NRS scores (Tables 3 and 4). However, patients homozygous for the major allele of rs10781468 required lower additional opioid doses than carriers of the minor allele under the dominant model (*p* = 0.025) (Table 4 and Figure 1). A similar trend was observed compared with heterozygotes in the genotypic model (Kruskal–Wallis test, *p* = 0.020; post hoc Bonferroni correction for major versus heterozygotes, *p* = 0.024) (Table 4), although NRS improvement after additional opioid administration did not differ significantly (Table 4). In the genotypic model, the association between rs10781468 and total opioid dose reached nominal significance (*p* = 0.045); however, none of the post hoc pairwise comparisons were significant (post hoc Bonferroni correction: major vs. heterozygotes, *p* = 0.06; heterozygotes vs. minor homozygotes, *p* = 0.24; major vs. minor homozygotes, *p* = 1.00). Regarding rs308049, no significant associations were observed with any evaluated outcomes.

**TABLE 3:** Associations of rs10781468 in the *GNAQ* gene and rs308049 in the *GNA11* gene polymorphisms with cancer pain and daily opioid dosages at the baseline assessment (Experiment 2). Opioid doses were converted to intravenous fentanyl equivalents and normalized to body weight. Pain intensity was measured using numeric rating scale (NRS). Jonckheere–Terpstra test was used for the additive model, Mann–Whitney *U* test was used for the recessive and dominant model, and Kruskal–Wallis test was used for the genotypic model.

Gene	SNPs	Genotype	*n* (female)	Baseline opioid (μg/kg/day)			Cancer pain intensity (NRS) at the baseline assessment		
				[Median (IQR)]	Model	*p*	[Median (IQR)]	Model	*p*
*GNAQ*	rs10781468 C>T	CC TC TT	35 (20) 39 (23) 15 (7)	0.275 (0–0.875) 0.489 (0.0332–2.46) 0.465 (0–0.858)	Additive	0.191	5 (4–7) 6 (5–8) 5 (4–6)	Additive	0.352
					Recessive	0.828		Recessive	0.405
					Dominant	0.193		Dominant	0.338
					Genotypic	0.323		Genotypic	0.280
*GNA11*	rs308049 C>T	CC TC TT	60 (32) 27 (16) 2 (2)	0.339 (0–1.23) 0.465 (0–1.37) 0.871 (0.498–1.24)	Additive	0.335	6 (4–7) 5 (4.5–7) 5 (4.5–5.5)	Additive	0.704
					Recessive	0.789		Recessive	0.556
					Dominant	0.692		Dominant	0.623
					Genotypic	0.905		Genotypic	0.777

**TABLE 4:** Associations of rs10781468 in the *GNAQ* gene and rs308049 in the *GNA11* gene polymorphisms with cancer pain and daily opioid dosages after therapeutic intervention (Experiment 2). Opioid doses were converted to intravenous fentanyl equivalents and normalized to body weight. Pain intensity was measured using the numeric rating scale (NRS). Jonckheere–Terpstra test was used for the additive model, Mann–Whitney *U* test was used for the recessive and dominant model, and Kruskal–Wallis test was used for the genotypic model.

Gene	SNPs	Genotype	*n* (female)	Additional opioid (μg/kg/day)			Total opioid (μg/kg/day)			Cancer pain intensity (NRS) after additional opioid administration			Cancer pain improvement by additional opioid administration (%)		
				[Median (IQR)]	Model	*p*	[Median (IQR)]	Model	*p*	[Median (IQR)]	Model	*p*	[Median (IQR)]	Model	*p*
*GNAQ*	rs10781468 C>T	CC TC TT	30 (6) 29 (17) 13 (6)	0.517 (0.245–0.857) 1.12 (0.467–2.16) 0.508 (0.279–0.1.40)	Additive	0.081	0.821 (0.441–1.55) 1.47 (0.645–4.22) 0.902 (0.536–2.05)	Additive	*0.146*	4 (3–5) 4 (3–5) 4 (3–5)	Additive	0.433	25 (0–50) 37.5 (25–62.5) 14.3 (0–50)	Additive	0.374
					Recessive	0.539		Recessive	*0.446*		Recessive	0.563		Recessive	0.318
					Dominant	0.025		Dominant	*0.063*		Dominant	0.926		Dominant	0.339
					Genotypic	0.020		Genotypic	*0.045*		Genotypic	0.789		Genotypic	0.201
*GNA11*	rs308049 C>T	CC TC TT	50 (25) 20 (12) 2 (2)	0.572 (0.343–1.22) 0.963 (0.265–1.47) 1.39 (0.756–2.02)	Additive	0.469	0.933 (0.587–2.23) 1.29 (0.435–2.38) 2.26 (1.25–3.26)	Additive	*0.566*	4 (3–5) 3 (2–5) 5 (5–5)	Additive	0.340	25 (0–57.1) 40 (0–60) −4.17 (−14.6–6.25)	Additive	0.505
					Recessive	0.851		Recessive	*0.694*		Recessive	0.323		Recessive	0.154
					Dominant	0.922		Dominant	*0.647*		Dominant	0.669		Dominant	0.878
					Genotypic	0.966		Genotypic	*0.773*		Genotypic	0.467		Genotypic	0.304

For the FDR‐adjusted results analyzed under each genetic model, the association between rs10781468 and additional opioid dose remained below the prespecified 20% FDR threshold under both the dominant and genotypic models (dominant model, *p* = 0.025, corresponding to FDR = 0.200; genotypic model, *p* = 0.020, corresponding to FDR = 0.200; Tables S4 and S5). In addition, sensitivity analysis adjusting for potential confounders (age, sex, and baseline opioid doses) demonstrated that rs10781468 remained significantly associated with total opioid dose under the genotypic model (*p* = 0.003; Tables S2 and S3).

## 4 Discussion

We conducted a two‐stage genetic analysis focusing on the relationship between G protein α subunits of GPCRs and both pain intensity and opioid sensitivity. The first exploratory stage involved postoperative pain, and the second confirmatory stage involved cancer pain. Analyses of the exploratory cohorts revealed that, for rs10781468 in the *GNAQ* gene, the minor allele was associated with reduced opioid consumption and an increased pain threshold, suggesting enhanced opioid sensitivity. In contrast, the minor allele of rs308049 in the *GNA11* gene appeared to be associated with increased opioid consumption. In the cancer pain cohort, patients homozygous for the major allele of rs10781468 required lower additional opioid doses to achieve comparable short‐term analgesia, suggesting reduced opioid sensitivity among carriers of the minor allele. rs308049 did not demonstrate any significant associations in this cohort. Overall, rs10781468 showed associations with opioid sensitivity in both the exploratory and confirmatory cohorts; however, the direction of the associations and the genetic models differed between cohorts. Because this study was hypothesis‐generating and included a limited number of participants, it was difficult to comprehensively account for multiple genotype comparisons and inheritance models. Accordingly, there is a substantial risk of false‐positive findings. Nevertheless, the present results, both with and without adjustment for confounding factors such as age, sex, and baseline opioid doses, may provide preliminary evidence that genetic polymorphisms in G protein α subunits are associated with pain intensity and opioid sensitivity.

GPCRs are the largest class of cell‐surface receptors and are ubiquitously distributed throughout the body [11]. They transduce a wide range of extracellular ligands to activate multiple intracellular signaling pathways, which regulate diverse physiological functions. G proteins are heterotrimeric complexes composed of Gα, Gβ, and Gγ subunits. Gα proteins are grouped into four subtypes (Gα_q/11_, Gα_12/13_, Gα_i/o_, and Gα_s_) and are encoded by the *GNAQ*, *GNA11*, *GNA12*, *GNA13*, *GNAI1*, *GNAO1*, and *GNAS* genes, respectively. Because distinct Gα subtypes largely determine the signaling properties of GPCRs, we focused on genetic polymorphisms in these Gα subtypes.

Several previous studies have demonstrated that opioid receptors generally transmit signals through the Gα_i/o_ family [12, 13]. Importantly, another line of studies reported that activation of Gα_q/11_ pathways contributes to enhanced opioid sensitivity [14]. A selective Gα_q/11_ pathway inhibitor not only exerted antinociceptive effects but also enhanced opioid analgesia [15]. Furthermore, evidence supports an intimate relationship between the Gα_q/11_ subtype and nociceptive pain processing. GPCRs for classical inflammatory mediators, such as bradykinin, histamine, and proteases, are coupled to the Gα_q_ subtype [16]. Mice with a nociceptor‐specific knockout of Gα_q/11_ exhibit reduced pain responses to mechanical stimuli in inflammatory pain models, as well as attenuated cold allodynia in neuropathic pain models [17]. Moreover, signaling through the orphan receptor GPR139 has been reported to attenuate opioid effects via Gα_q/11_ [18]. Notably, experiments using knockout mice demonstrated that Gα_q_, rather than Gα_11_, plays a predominant role in nociceptor sensitization [19]. In addition, mice lacking the Gα_q_‐coupled P2Y1 receptor or treated with a P2Y1 antagonist exhibited reduced nocifensive responses to noxious heat in inflammatory pain models [20]. Taken together, numerous animal studies suggest that Gα_q_ modulates opioid sensitivity and pain intensity. Human studies indicate that opioid sensitivity and pain intensity depend on multiple interacting variables. Previous reports have identified factors related to opioid analgesics themselves, such as dose and metabolism, as well as subject‐specific variables, including age, sex, and type of pain [21]. Population pharmacokinetic studies have demonstrated that healthy females achieve 20%–35% higher plasma opioid area under the curve than healthy males at identical weight‐based doses. This sex difference may be attenuated by age‐related changes in drug metabolism [21]. Without incorporating these interacting variables in the primary analyses, we focused on the relationships among genetic polymorphisms of Gα subtypes, opioid sensitivity, and pain intensity. Additionally, in sensitivity analyses, we applied multivariable models adjusting for covariates such as age, sex, and baseline opioid dose. Consequently, rs10781468 remained significantly associated under the genotypic model, suggesting that this SNP in the *GNAQ* gene may be associated with opioid sensitivity and inflammatory pain severity. However, the possibility of false‐positive findings should be considered.

Regarding genetic polymorphisms in G protein α subunits, previous studies have demonstrated that various variants are associated with different phenotypes [4]. However, in the Human Pain Genetics Database (HPGDB), a comprehensive resource of pain‐associated genes, neither rs10781468 nor its host gene, *GNAQ*, is listed [22]. The observation that the direction of the effect of the rs10781468 risk allele on opioid responsiveness differed between the exploratory and confirmatory stages raises the question of why the GNAQ gene has not been previously documented in the HPGDB. First, it is important to note that the nature of pain in the populations analyzed in the two stages differed. Postoperative pain is primarily an acute inflammatory condition, whereas cancer pain is a complex and persistent state that includes both nociceptive and neuropathic components and may worsen over time. Such pathophysiological differences may partly explain the discordant direction of opioid sensitivity associated with rs10781468. In addition, as noted above, the Gα_q_ subunit is generally considered to enhance pain signaling; however, some reports suggest that Gα_q_‐related signaling may contribute to reduced opioid sensitivity under conditions of repeated opioid exposure [23]. In other words, the effect of rs10781468 may manifest through both its influence on pain processing via Gα_q_ signaling and its potential role in tolerance development or opioid‐induced hyperalgesia during repeated opioid exposure. This perspective may provide a coherent explanation for the observed differences in opioid responsiveness across populations in the present study.

However, rs10781468 in the *GNAQ* gene is an intronic variant, and to our knowledge, no reports have described its functional consequences. Therefore, the involvement of this Gα_q_ genetic polymorphism in opioid sensitivity and pain intensity should be interpreted with caution and validated in future studies, with careful consideration of the following limitations.

First, the number of enrolled patients was relatively small. Because the present study was exploratory in nature, the analyses did not incorporate formal correction for multiple comparisons across SNPs, phenotypes, and inheritance models. Accordingly, the reported *p*‐values are presented without adjustment for multiplicity and should be interpreted as hypothesis‐generating. Second, divergent results in opioid sensitivity were observed between the first and second stages. These discrepancies may reflect differences in pain type (postoperative pain vs. cancer pain). Future large‐scale studies should focus on a single, homogeneous pain type. Because a consistent association with opioid sensitivity for the *GNAQ* genetic polymorphism was observed in the two postoperative pain cohorts, postoperative pain may be a more suitable target for future validation studies. Third, owing to the observational study design conducted in routine clinical settings, the opioid administration protocol was not standardized, and the dose and timing of opioid use were determined at the clinicians' discretion. In addition, the three cohorts differed in the assessment of pain phenotypes. Therefore, further studies with larger sample sizes and prospective designs are required to standardize opioid administration protocols and unify pain phenotype assessment to distinguish reduced opioid sensitivity from pain exacerbation. Fourth, we could not completely exclude the potential influence of somatic mutations, particularly in the cancer pain cohort. Although genotyping was performed using peripheral blood–derived DNA rather than tumor tissue and no overt mosaicism was observed, detailed tumor histology was not available. Therefore, the possibility of low‐level somatic variation or clonal hematopoiesis influencing the results cannot be entirely excluded. Finally, this study did not primarily incorporate multivariable models adjusting for covariates such as age, sex, or baseline opioid dose. Preliminary sensitivity analyses indicated that these variables were not strongly associated with the primary outcomes in our sample, and adjustment did not meaningfully alter the observed associations. Nevertheless, the absence of comprehensive adjustment limits causal interpretation, and future studies with larger samples and more extensive covariate data are warranted to validate these findings.

Our findings suggest that rs10781468 in the *GNAQ* gene may be associated with the amount of additional opioid required to achieve comparable short‐term analgesia. Although there is a substantial risk of false‐positive findings and the above limitations must be considered, this observation may serve as a preliminary hypothesis for future studies.

## Author Contributions

Study conception: D.N., K.I., M.S.; Study design: D.N., M.H., T.I., K.I., M.S.; Data collection: T.M., M.T., H.N., S.Y., A.K., K.‐I.F.; Data analysis: H.O., D.N., K.I.; Data interpretation: H.O., R.T., Y.Y., M.K., K.U., M.S.; Drafting of article: H.O., D.N., M.S.; Reviewing paper: all authors; Approval of the final version: all authors.

## Funding

This study was funded by the Japanese Ministry of Health, Labor, and Welfare Science Research Grant (Grant number: H21‐Cancer‐011), JSPS KAKENHI Grant Number (22H04922 [AdAMS]) and Japan Agency for Medical Research and Development (Grant number: 23ek0610028h0002).

## Ethics Statement

This study was approved by the Institutional Review Board of each participating hospital (G2804), and all participants provided written informed consent. This study was registered in the University Medical Information Network (UMIN; trial ID: UMIN000008595).

## Consent

Written informed consent was obtained from all participants at each hospital.

## Conflicts of Interest

The authors declare no conflicts of interest.

## Supporting information


**Table S1:** Results of the sensitivity analyses for associations of rs10781468 in the *GNAQ* gene and rs308049 in the *GNA11* gene polymorphisms with clinical measurements in patients receiving laparoscopic‐assisted colectomy and mandibular sagittal split ramus osteotomy, respectively. Multivariate linear regression analysis was performed using age and sex as covariates. *p* < 0.05 is considered as significant, but multiplicity is not considered. N.A., not applicable.
**Table S2:** Results of the sensitivity analyses for associations of rs10781468 in the *GNAQ* gene and rs308049 in the *GNA11* gene polymorphisms with cancer pain and daily opioid dosages at the baseline assessment. Multivariate linear regression analysis was performed using age and sex as covariates. *p* < 0.05 is considered as significant, but multiplicity is not considered.
**Table S3:** Results of the sensitivity analyses for associations of rs10781468 in the *GNAQ* gene and rs308049 in the *GNA11* gene polymorphisms with cancer pain and daily opioid dose after therapeutic intervention. Multivariate linear regression analysis was performed using age, sex, and baseline opioid dose as covariates. *p* < 0.05 is considered as significant, but multiplicity is not considered.
**Table S4:** Results of FDR correction using the Benjamini‐Hochberg step‐up method for pretherapeutic intervention endpoints in the cancer pain cohort regarding rs10781468 in the *GNAQ* gene. *q* ≤ 0.20 is considered as significant.
**Table S5:** Results of FDR correction using the Benjamini‐Hochberg step‐up method for posttherapeutic intervention endpoints in the cancer pain cohort regarding rs10781468 in the *GNAQ* gene. *q* ≤ 0.20 is considered as significant.

## Data Availability

The data supporting the findings of this study are not publicly available due to ethical and regulatory restrictions. Because the study protocol approved by the institutional review board did not include provisions for public data release, participants were not informed during the informed consent process that their genetic polymorphism data might be made publicly available, and consent for public data sharing was therefore not obtained. Consequently, public disclosure of the data would be outside the scope of the approved study protocol and inconsistent with the participants' consent.
