# Supplementary material for: The rs10781468 Genetic Polymorphism of the Gαq (GNAQ) Gene Is Associated With Responsiveness to Opioid Analgesics in Patients With Postoperative and Cancer Pain: An Exploratory Study
Source: Neuropsychopharmacol Rep. 2026 Jun 30;46(3):e70148. doi: 10.1002/npr2.70148 (PMC13316446; doi:10.1002/npr2.70148)
Supplement: Supplementary file 1 — Table S1: Results of the sensitivity analyses for associations of rs10781468 in the GNAQ gene and rs308049 in the GNA11 gene polymorphisms with clinical measurements in patients receiving laparoscopic‐assisted colectomy and mandibular sagittal split ramus osteotomy, respectively. Multivariate linear regression analysis was performed using age and sex as covariates. p < 0.05 is considered as significant, but multiplicity is not considered. N.A., not applicable. Table S2: Results of the sensitivity analyses for associations of rs10781468 in the GNAQ gene and rs308049 in the GNA11 gene polymorphisms with cancer pain and daily opioid dosages at the baseline assessment. Multivariate linear regression analysis was performed using age and sex as covariates. p < 0.05 is considered as significant, but multiplicity is not considered. Table S3: Results of the sensitivity analyses for associations of rs10781468 in the GNAQ gene and rs308049 in the GNA11 gene polymorphisms with cancer pain and daily opioid dose after therapeutic intervention. Multivariate linear regression analysis was performed using age, sex, and baseline opioid dose as covariates. p < 0.05 is considered as significant, but multiplicity is not considered. Table S4: Results of FDR correction using the Benjamini‐Hochberg step‐up method for pretherapeutic intervention endpoints in the cancer pain cohort regarding rs10781468 in the GNAQ gene. q ≤ 0.20 is considered as significant. Table S5: Results of FDR correction using the Benjamini‐Hochberg step‐up method for posttherapeutic intervention endpoints in the cancer pain cohort regarding rs10781468 in the GNAQ gene. q ≤ 0.20 is considered as significant. [file NPR2-46-e70148-s001.docx]

| Operation type | Gene | SNPs | Prolongation of pain latencies immediately after opioid administration | | | Postoperative opioid consumption (μg /kg) | | | Postoperative pain intensity (VAS) | | |
| --- | --- | --- | --- | --- | --- | --- | --- | --- | --- | --- | --- |
|  |  |  | Additive | Recessive | Dominant | Additive | Recessive | Dominant | Additive | Recessive | Dominant |
| Laparoscopy-assisted colectomy (LAC)cohort | *GNAQ* | rs10781468 C>T | *N.A.* | *N.A.* | *N.A.* | *0.209* | *0.096* | *0.285* | *0.081* | *0.061* | *0.536* |
|  | *GNA11* | rs308049 C>T | *N.A.* | *N.A.-* | *N.A.* | *0.084* | *0.044* | *0.146* | *0.744* | *0.502* | *0.843* |
| Mandibular sagittal split ramus osteotomy (SSRO) cohort | *GNAQ* | rs10781468 C>T | *0.026* | *0.018* | *0.047* | 0.172 | 0.064 | 0.367 | *0.305* | *0.150* | *0.964* |
|  | *GNA11* | rs308049 C>T | *0.678* | *0.529* | *0.656* | 0.105 | 0.034 | 0.734 | *0.847* | *0.565* | *0.915* |

Table S1. Results of the sensitivity analyses for associations of rs10781468 in the *GNAQ* gene and rs308049 in the *GNA11* gene polymorphisms with clinical measurements in patients receiving laparoscopic-assisted colectomy and mandibular sagittal split ramus osteotomy, respectively. Multivariate linear regression analysis was performed using age and sex as covariates. *p* < 0.05 is considered as significant, but multiplicity is not considered. N.A. = not applicable

| Gene | SNPs | Baseline opioid (μg /kg/day) | | | | Cancer pain intensity (NRS)  at the baseline assessment | | | |
| --- | --- | --- | --- | --- | --- | --- | --- | --- | --- |
|  |  | Additive | Recessive | Dominant | Genotypic | Additive | Recessive | Dominant | Genotypic |
| *GNAQ* | rs10781468 C>T | *0.481* | *0.681* | *0.176* | *0.188* | *0.489* | *0.500* | *0.118* | *0.150* |
| *GNA11* | rs308049 C>T | *0.943* | *0.716* | *0.847* | *0.942* | *0.673* | *0.556* | *0.780* | *0.826* |

Table S2. Results of the sensitivity analyses for associations of rs10781468 in the *GNAQ* gene and rs308049 in the *GNA11* gene polymorphisms with cancer pain and daily opioid dosages at the baseline assessment. Multivariate linear regression analysis was performed using age and sex as covariates. *p* < 0.05 is considered as significant, but multiplicity is not considered.

| Gene | SNPs | Additional opioid dose (μg/kg/day) | | | | Total opioid dose (μg/kg/day) | | | | Cancer pain intensity (NRS)  after additional opioid administration | | | | Cancer pain improvement  by additional opioid administration (%) | | | |
| --- | --- | --- | --- | --- | --- | --- | --- | --- | --- | --- | --- | --- | --- | --- | --- | --- | --- |
|  |  | Additive | Recessive | Dominant | Genotypic | Additive | Recessive | Dominant | Genotypic | Additive | Recessive | Dominant | Genotypic | Additive | Recessive | Dominant | Genotypic |
| *GNAQ* | rs10781468 C>T | 0.936 | 0.609 | 0.776 | 0.142 | 0.789 | 0.540 | 0.936 | 0.003 | 0.614 | 0.332 | 0.997 | 0.613 | 0.953 | 0.360 | 0.415 | 0.252 |
| *GNA11* | rs308049 C>T | 0.989 | 0.948 | 0.994 | 0.936 | 0.839 | 0.858 | 0.867 | 0.769 | 0.740 | 0.253 | 0.979 | 0.718 | 0.572 | 0.060 | 0.988 | 0.260 |

Table S3. Results of the sensitivity analyses for associations of rs10781468 in the *GNAQ* gene and rs308049 in the *GNA11* gene polymorphisms with cancer pain and daily opioid dose after therapeutic intervention. Multivariate linear regression analysis was performed using age, sex, and baseline opioid dose as covariates. *p* < 0.05 is considered as significant, but multiplicity is not considered.

| Rank | Phenotype | Model | *p*-value | *q*-value |
| --- | --- | --- | --- | --- |
| 1 | Baseline opioid dose | Additive | 0.191 | 0.828 |
| 2 | Baseline opioid dose | Dominant | 0.193 | 0.828 |
| 3 | pre-NRS | Genotypic | 0.280 | 0.828 |
| 4 | Baseline opioid dose | Genotypic | 0.323 | 0.828 |
| 5 | pre-NRS | Dominant | 0.338 | 0.828 |
| 6 | pre-NRS | Additive | 0.352 | 0.828 |
| 7 | pre-NRS | Recessive | 0.405 | 0.828 |
| 8 | Baseline opioid dose | Recessive | 0.828 | 0.828 |

Table S4. Results of FDR correction using the Benjamini-Hochberg step-up method for pre-therapeutic intervention endpoints in the cancer pain cohort regarding rs10781468 in the *GNAQ* gene. q ≤ 0.20 is considered as significant.

| Rank | Phenotype | Model | *p*-value | *q*-value |
| --- | --- | --- | --- | --- |
| 1 | Additional opioid dose | Genotypic | 0.020 | 0.200 |
| 2 | Additional opioid dose | Dominant | 0.025 | 0.200 |
| 3 | Total opioid dose | Genotypic | 0.045 | 0.212 |
| 4 | Total opioid dose | Dominant | 0.053 | 0.212 |
| 5 | Additional opioid dose | Additive | 0.081 | 0.259 |
| 6 | Total opioid dose | Additive | 0.146 | 0.389 |
| 7 | NRS improvement | Genotypic | 0.201 | 0.459 |
| 8 | NRS improvement | Recessive | 0.318 | 0.595 |
| 9 | NRS improvement | Dominant | 0.339 | 0.595 |
| 10 | NRS improvement | Additive | 0.374 | 0.595 |
| 11 | post-NRS | Additive | 0.433 | 0.595 |
| 12 | Total opioid dose | Recessive | 0.446 | 0.595 |
| 13 | Additional opioid dose | Recessive | 0.539 | 0.643 |
| 14 | post-NRS | Recessive | 0.563 | 0.643 |
| 15 | post-NRS | Genotypic | 0.789 | 0.842 |
| 16 | post-NRS | Dominant | 0.926 | 0.926 |

Table S5. Results of FDR correction using the Benjamini-Hochberg step-up method for post-therapeutic intervention endpoints in the cancer pain cohort regarding rs10781468 in the *GNAQ* gene. q ≤ 0.20 is considered as significant.
